# Biodefense and emergency use authorization: different originations, purposes, and evolutionary paths of institutions in the United States and South Korea

**DOI:** 10.1186/s12992-022-00895-5

**Published:** 2022-12-05

**Authors:** HyunJung Kim

**Affiliations:** Barun ICT Research Center, Yonsei University, 50 Yonsei-ro, Seodaemun-gu, Seoul, 03722 South Korea

**Keywords:** Biodefense, Emergency-use-authorization, Historical institutionalism, Homeland security, Health security, Public health emergency, Disease containment

## Abstract

**Background:**

Emergency-use-authorization (EUA) is the representative biodefense policy that allows the use of unlicensed medical countermeasures or off-label use of approved medical countermeasures in response to public health emergencies. This article aims to determine why the EUA policies of the United States and South Korea produced drastically different outcomes during the COVID-19 pandemic, and how these outcomes were determined by the originations and evolutionary paths of the two policies.

**Method:**

Historical institutionalism (HI) explains institutional changes—that is, how the institution is born and how it evolves—based on the concept of path dependency. However, the HI analytical narratives remain at the meso level of analysis in the context of structure and agency. This article discusses domestic and policy-level factors related to the origination of the biodefense institutions in the United States and South Korea using policy-learning concepts with the Event-related Policy Change Model.

**Results:**

The 2001 anthrax letter attack (Amerithrax) and the 2015 Middle East Respiratory Syndrome (MERS) outbreak prompted the establishment of biodefense institutions in the United States and South Korea, respectively. Due to the different departure points and the mechanism of path dependency, the two countries’ EUAs evolved in different ways—the United States EUA reinforced the Post-Exposure Prophylaxis (PEP) function, while the South Korea EUA strengthened the Non-Pharmaceutical Intervention (NPI) function.

**Conclusions:**

The evolution and outcomes of the two EUAs are different because both policies were born out of different needs. The United States EUA is primarily oriented toward protecting homeland security against CBRN (chemical, biological, radiological, and nuclear) threats, whereas the South Korea EUA is specifically designed for disease prevention against infectious disease outbreak.

## Introduction

The emergency use of unapproved medical countermeasures (MCMs) is an innovative policy enabling the use of MCMs^1^ not yet licensed by the domestic drug approval system for use in public health emergencies. Since the COVID-19 pandemic, the emergency use of unapproved MCMs has been continuously cited in the media and academia. Prior to the COVID-19 pandemic, the United States (US) and South Korea were the only two countries that had already developed their respective Emergency Use Authorization (EUA) policies to allow the distribution and use of investigational MCMs or to allow off-label use of approved MCMs in response to public health emergencies.^2^ In response to the current COVID-19 pandemic, both the US and South Korea issued EUAs for COVID-19 in-vitro diagnostic (IVD) kits on 4 February 2020. However, the continued lack of COVID-19 testing in the US delayed timely infection intervention, a clear failure when compared to the massive volume of suspected case testing carried out in South Korea [1].

The main question that this study seeks an answer to is: why did the EUAs of the US and South Korea yield substantially different outcomes in terms of COVID-19 testing volume? The US EUA was unable to support and facilitate large-scale COVID-19 testing during the early phase of the pandemic, whereas the Korean EUA quickly facilitated nation-wide COVID-19 testing. What exactly is different about the EUA policy approaches between the two countries? Expanding on the existing historical institutionalism (HI) literature, this article identifies the critical junctures in the US and South Korea that led to the emergence of different policy domains that shaped the EUA policy of each country. Specifically, this study examines three dimensions of both nations’ EUA policies—origin, purpose, and features. The concept of path dependency can explain the evolutionary pattern of the US and South Korean EUA policies, both of which have gradually expanded in scope to include other potential threats in subsequent legislations. Theoretical debate in the HI school of thought between Hall and Taylor vs. Hay and Wincott provides us deeper insights about the policy evolutions fully incorporating new institutionalism in the context of the relationship between structure and agency [2–4]. Beyond the “latent structuralism” title, many HI scholars have studied the role of agency—the attributes of power and idea—when it comes to the mutability of institutions. However, the analytical framework of HI still has theoretical limitations as it allows only a meso-level analysis of institutional changes [5].

This article breaks away from this limitation by applying the Thomas Birkland’s Event-Related Policy Change Model. Birkland examined the event-related policy shift process at the domestic level by considering aspects such as focusing event, group mobilization, and addenda-setting. His Event-Related Policy Change Model explains how lessons from catastrophic events change institutions or policies by shaping political behaviors and domestic coalitions. Making up for the HI tenets, the Birkland model provides a detailed theoretical lens to understand how policy actually changes at the domestic level.

This article explores the adoption, revision, and evolution of EUA policies in the US versus South Korea taking into account that both policies have different originations, purposes, and evolutionary pathways. EUA policies in both nations were legislated with different origins and purposes depending on the biodefense institutions in each country. The US EUA was legislated after the 2001 anthrax attacks (Amerithrax) and underwent multiple revisions over time. The Korean EUA was legislated after the 2015 Middle East Respiratory Syndrome (MERS) outbreak, and radiation exposure was later added to the list of targeted threats. It is worth noting that a homeland security centric biodefense institution in the US and a disease containment centric biodefense institution in South Korea have been reinforced by subsequent policy revisions, instead of being replaced.

Much of the HI literature emphasizes the role of endogenous factors (e.g., political behaviors), and yet strong descriptive studies explaining how institutional changes really work are missing. Hence, this article reviews the latest discussions on dynamics of exogenous or endogenous factors and how they mutually shape each other, leading to institutional changes. By integrating the HI narratives with Birkland’s model, this article provides a detailed descriptive study illustrating how endogenous factors (agency) come into play when an institution faces exogenous shocks (structural shifts). This article includes case studies of the US and South Korea illustrating in detail how institutional changes are influenced by EUA policy; in other words, how both countries adopted and revised their biodefense institutions in different ways.

### Biodefense as an institution

An institution is often defined as an organizational structure of the polity consisting of formal or informal procedures, routines, norms, and conventions, that shape political behaviors and outcomes of political processes [2, 6, 7]. In this context, biodefense can be considered an institution consisting of various policies and organizations that govern the behaviors of a set of individuals within a given society. For example, the US has regularly published a National Biodefense Strategy, which provides a framework for orchestrating diverse biodefense activities across federal departments and agencies in order to protect American lives from biological threats [8]. Biodefense also includes the implementation of various activities related to counter-bioterrorism and biological warfare, arms control and nonproliferation, bio-surveillance, emergency preparedness, and MCM development. Thus, biodefense entails actions designed to counter biological threats; reduce risks; and prepare for, respond to, and recover from bio-incidents [9]. The field of biodefense is thus treated as an institution where US national security concerns arouse political leaders to take actions for adopting, revising, or withdrawing biodefense policies [10].

There are multiple theories and models that seek to explain how policies, especially major policy changes, emerge. Echoing the tenets of historical institutionalism, this article posits that biodefense as an institution governs the behavior of people and shapes unique political objectives. The specific focus of this article is on policy changes (EUA policy) in the field of biodefense. An EUA is policy that allows large-scale distribution of investigational or new MCMs at the national level, regardless of potential adverse effects, to deal with a public health emergency. As seeing Table 1, EUA policy represents the features of biodefense institutions in each country.

**Table 1:** Comparing key characteristics of US and Korean biodefense

Characteristics	United States	South Korean
**Origination of the Policy**	2001 anthrax letter attack	2015 MERS outbreak
**Purpose of the Policy**	Preparedness & Response	Detection & Diagnosis
**Target of the Policy**	CBRN	Infectious Diseases
**Revised Target**	All-Hazards	Radiation Exposure

The primary research methodology used in this article is case study. Case study is a type of qualitative research that examines complex phenomena in the natural setting to increase understanding of them [11]. Especially, Case study is a “good part of the backbone of policy analysis and research” [12]. The cases of US and South Korea were selected for comparative study because prior to the COVID-19 pandemic, these were the only two countries with EUA policies established by legislation. After the COVID-19 outbreak, many other countries (e.g., United Kingdom) adopted EUA or EUA-like policies to temporarily authorize the supply of new COVID-19 vaccines. This article conducted a comparative study of the US and South Korean EUA policies in order to determine how their respective originations, purposes, and evolutionary paths have shaped the two countries’ biodefense institutions. Such an analysis can contribute to building the theoretical background for further research on the following questions: (1) Why did the South Korean EUA successfully accomplish mass-testing by authorizing in-vitro diagnostic kits but substantially delayed the authorization of mass administration of new investigational COVID-19 vaccines? (2) Why was the US EUA less effective in quickly expanding the COVID-19 testing campaigns, but promptly allowed mass administration of new investigational COVID-19 vaccines?

Building upon the idea of diverse policy approaches, the main question under focus in this article is: why do the EUA policies of the US and South Korea operate so differently? In general, historical institutionalism (HI) provides a theoretical lens that focuses on institutional origins and changing patterns with the assumption that institutions come, in a meaningful sense, from the past. Based on structural-functionalist tenets, HI accounts for institutional origins and changes in the language of *critical juncture*, which is a decisive moment of innovation caused by crises (exogenous shocks) such as a revolution, war, or regime change. Critical juncture is referred to as a period of significant change which typically shapes the national political arena in different countries in distinct ways [2, 6]. In this view, the 2001 anthrax attacks and the 2015 MERS outbreak were critical junctures in the US and South Korea, respectively. Indeed, the US EUA was legislated after the 2001 anthrax attacks and the Korean EUA was reformed after the 2015 MERS outbreak.

Due to the different critical junctures—the 2001 anthrax attacks and the 2015 MERS outbreak—the institutional outcomes (set of processes, i.e., rules, procedures, or policies) of the two countries evolved in different directions. The US EUA was born out of the need to strengthen homeland security while the Korean EUA was created for disease containment [13]. In general, institutional outcomes are shaped by policy purpose and process, and are purely a consequence of domestic politics and the political behaviors of agents such as coalitions or interest groups. Many HI studies point out the limitation of structuralist HI narratives focusing solely on the results of exogenous factors. These scholars understand institutions as the products of agency, rather than constraints.

### Structure and agency: driving forces to lead institutional changes

The US EUA as well as the South Korean EUA underwent multiple revisions over time since their adoption. It is important to note that both policies were revised in response to subsequent issues and events. For example, due to events such as Hurricane Katrina and H1N1 influenza, the purview of the US EUA expanded from bioterrorism to all-hazards. The purview of the Korean EUA also expanded from infectious diseases to radioactive contamination. Although both policies have expanded, the core principles of both EUAs have been kept intact and even strengthened over the course of policy revisions. For example, post-exposure prophylaxis (PEP) is the policy core of the US EUA as the policy’s aim is to support mass-distribution of vaccines in the event of CBRN terrorism (homeland security purpose), while non-pharmaceutical intervention (NPI) is the policy core of the Korean EUA so as to support a mass-testing campaign in the case of infectious disease outbreak (disease containment purpose). Every time the EUAs of both countries are revised, the revisions reinforce the policy cores (PEP and NPI, respectively). In theory, these patterns of institutional innovation often rely on or share the same pathway of development with the previous innovation; this is called *path dependency.*

Basically, critical junctures are the starting points of many path-dependent processes, and path dependence is a crucial causal mechanism according to HI scholars [14]. The main logical foundation of path dependence is “self-reinforcement,” meaning that social systems tend to converge on a single path which is the product of an arbitrary initial decision or interaction that leads to self-reinforcing patterns [15]. The self-reinforcing nature of institutional innovation can be explained further through the notion of *punctuated equilibria*, where brief and sporadic moments, as critical junctures, become triggers of institutional change by collapsing existing institutions or providing actors with the opportunity to select a different path [16, 17]. A distinguished biodefense scholar, Richard Danzig, points out that the development of US biodefense policies has followed a pattern of “punctuated evolution,” where changes only occur when an exogenous shock forces decision-makers to take actions [10]. In the same vein, the US federal biodefense policy and MCM development, particularly coverage of pediatric populations, was strengthened by legislations following Hurricane Katrina [18]. The tenets of punctuated evolution can explain why institutional changes have sustained the policy core of EUA policy in each country, thereby establishing an evolutionary pathway. This policy evolution implies that once a new policy domain is accepted or institutionalized in a society, the society is likely to pile up new emerging domains neatly atop the previous one rather than replacing old with new, in essence similar to path-dependent innovations. However, the current theoretical framework of HI is still limited to a detailed account explaining the effectiveness of environmental shifts influencing the role of endogenous factors [19–21].

The purview and explanatory power of the punctuated equilibria framework long remained in the structural-functionalist context, which discussed abstract causality between exogenous factors (environmental shifts or shocks) and a pattern of institutional change. In other words, structural-functionalist narratives through critical juncture and path dependency are eligible for explaining the originations and directions of institutional change to a larger context (more than meso-level), but are less likely to explain the mechanisms at play in domestic politics (e.g., policy community) and how endogenous forces (e.g., agency) lead to subsequent institutional changes. The role of agents in the course of endogenous institutional change becomes central to HI discussions in addressing that human (agencies) enact institutions, and they likewise transform institutions in response to environmental changes, and thus, institutional outcomes can change over time [19, 22, 23]. Back into the debate between Hall and Taylor vs. Hay and Wincott, the crux of the matter is how we define and draw the dynamic of structure and agency in terms of institutional changes. Scholars emphasize the role of the wider meta institutional context in addressing agents and structures are mutually shaping each other over time [24]. “Meta institutional” analysis examines institutional changes from various angles such as structural context, crises, and wider power context or policy context [25]. Moreover, to strengthen connectivity between exogenous and endogenous factors, Slater and Simmons [26] highlight the role of the “antecedent condition,” which is a condition preceding a critical juncture, in determining the precise causal and non-causal status of institutional changes. Soifer [27] also emphasizes preconditions, whether permissive or productive, of critical junctures to precisely analyze the causality of institutional changes.

## Method: event-related policy change model

### Coherent explanatory power to Back up historical institutionalism

The new HI literatures give more attention to endogenous factors but still remain limited to meso-level analysis which can hardly explain “how it actually changed?” To compensate for the lack of adequate descriptive power in explaining how political behaviors are shaped by the interaction of structure and agency, some scholars borrow the concept of policy-learning [28–30]. The learning process by which participants use information and knowledge to develop, test, and refine their beliefs becomes the center of academic debates [31–34].

**Fig. 1:**
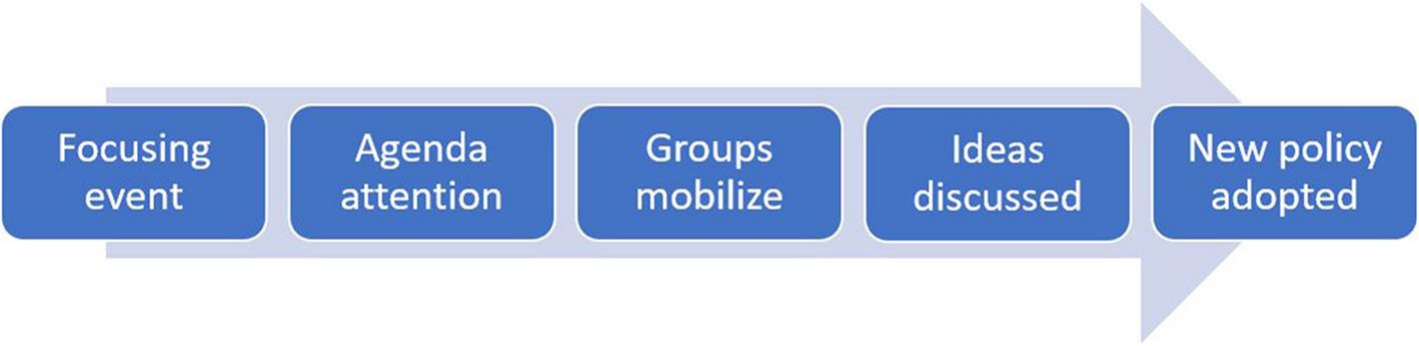
Event-Related Policy Adopting/Changing Process

Thomas Birkland developed a policy learning model illustrating how a society learns lessons from focusing events (exogenous shocks) that facilitate policy changes within the society (seeing Fig. 1). He emphasizes the emergence of policy domain as a learning result from focusing event. The policy domain is generally defined and studied as a component of the political system that is organized around substantive issues. Policy domains are largely socially constructed, varying with issues and politics, which leads to legislative enactment of major policy change [35]. Therefore, groups of congregated agencies (namely, society or the public) and the dynamic interactions among agencies such as conflicts and contests between agents (e.g., turf war) become the centers of institutional change. Birkland’s model contributes to explaining the role of exogenous shocks (critical junctures or focusing events) as facilitators for endogenous dynamics, which can increase public attention on a problem and lead to the emergence of a new policy domain resulting in policy changes [36].

This model effectively demonstrates the mutuality between structure and agent in addressing that once an exogenous shock triggers the emergence of new political agenda and political behaviors of agents, the endogenous forces lead to institutional changes; but the newly mobilized endogenous factors also perform a vital role of sustaining and reinforcing policy cores. Therefore, from the time a group is mobilized after a crisis, the group remains in the society and plays a significant role in strengthening policy cores (e.g., PEP and NPI) owing to the mechanism of self-reinforcement. The case study in the next chapter illustrates how a homeland security group was mobilized in the US after the 2001 Amerithrax and why the policy core (PEP) of the US EUA was strengthened by two policy revisions after Hurricane Katrina (2005) and the H1N1 pandemic (2009). Similarly, epidemiologists were mobilized in Korea after the 2015 MERS outbreak, and the policy core (NPI) of the Korean EUA was strengthened by a revision made due to the 2018 trade war with Japan.

#### Case study of the United States

##### Twin focusing events (9/11 and Amerithrax) and a new agenda (counterterrorism)

The adoption of the homeland security policy domain dominated all areas and fields of the post-9/11 movement in the United States. The former Secretary of DHS under the Obama administration, Janet Napolitano, views, in retrospect, that Americans in 2001 - including both ordinary citizens and those in the highest levels of the US government - were seized by a national sense of paranoia and dread of terrorism [37]. The emergence of the homeland security domain in parallel with expanding counter-terrorism efforts mobilized the homeland security group. President G.W. Bush issued Executive Order 13228 on 8 October 2001, which established the Office of Homeland Security within the Executive Office of the President. Executive Order 13228 called for the coordination of US national efforts against terrorism threats and, consequently, contributed to the mobilization of the homeland security group.

Along with the increasing concerns of conventional terrorism threats emerging from 9/11, the 2001 anthrax letter attacks added a new concern of terrorists exploiting weapons of mass destruction (WMDs), especially with regards to biological weapons. Counterterrorism and WMD nonproliferation became the top priority for US policy agendas following 9/11 and Amerithrax in 2001. To protect the US homeland and population, it was deemed necessary to recognize emerging CBRN terrorism as a potential new type of public health threat. On 12 October 2001, Vice President Dick Cheney stated that it is “reasonable” to assume the anthrax attacks were linked to the 9/11 terrorist attacks, because al-Qaeda-trained operatives know “how to deploy and use these kinds of substances [weaponizable biological and chemical materials]” [38]. At a 15 October 2001 press conference, President George W. Bush stated that “there may be some possible link” between the anthrax-contained envelopes and Osama bin Laden, adding “I wouldn’t put it past him” [39].

Accompanying the increasingly political narratives concerning CBRN terrorism threats, the majority of the post-Amerithrax evaluations and investigations held critical reviews for all levels of the US public health emergency system and made policy recommendations for what should be done in such future scenarios with focuses on preparedness and response. For example, the US Defense Threat Reduction Agency (DTRA) and Center for Strategic and International Studies (CSIS) published a joint post-event analysis report. The US DTRA-CSIS report concludes that the 2001 anthrax letter attacks, along with the September 11th attacks, forced the United States to confront new threats –terrorism within the homeland and the proliferation of WMDs - thus assigning public health as a key element to US defense [40].

##### Homeland security group mobilized

The National Commission on Terrorist Attacks Upon the United States (also known as the 9/11 Commission) was established on 27 November 2002 by Public Law 107–306. The law directed the 9/11 Commission to investigate “facts and circumstances relating to the terrorist attacks of September 11, 2001,” including those relating to intelligence agencies, law enforcement agencies, diplomacy, immigration issues and border control, the flow of assets to terrorist organizations commercial aviation, the role of congressional oversight and resource allocation and other areas determined relevant by the Commission [39]. The post-9/11 counterterrorism efforts expanded in scope to include the non-traditional counter-terrorism disciplines and began to consolidate them to one name: homeland security. Finally, the Homeland Security Act of 2002 was enacted on 25 November 2002, which authorized the establishment of the US Department of Homeland Security (DHS). The Homeland Security Act is a historical milestone of US national security that mobilized resources and efforts across all levels of government to deal with terrorism threats. The Homeland Security Act of 2002 brought many responsibilities for public health preparedness and response within one department (DHS), which was composed of 180,000 personnel from 22 federal organizations.

The newly formed homeland security group embraced biodefense topics since its origin following Amerithrax. In other words, biodefense became one of core subjects of counterterrorism through homeland security efforts. On 12 June 2002, the Public Health Security and Bioterrorism Preparedness and Response Act (PL 107–188, 2002; also known as the Bioterrorism Act [41]) was signed into effect. The purpose of this law was to strengthen national preparedness for bioterrorism and other public health emergencies, giving much more weigh to security benefits over public health benefits. One of the most notable biodefense inventions created by the Bioterrorism Act was the concept of “Select Agents” to tighten control and restrict access to certain dangerous biological agents and toxins. Also, it established the Strategic National Stockpile (SNS) to maintain a stockpile of medical countermeasures and necessary supplies in the event of bioterrorism or another public health emergency [42].

Both the Public Health Security and Bioterrorism Preparedness and Response Act and Homeland Security Act of 2002 solidified the urgency of CBRN terrorism threats as the post-9/11 and post-Amerithrax homeland security domain overtook public health domains. The United States government immediately reacted to the September 11th and the anthrax letter attacks as one event, which lumped public health issues into homeland security benefits. The US General Accounting Office (GAO) released a post-Amerithrax evaluation report. Written for the US Senate, that emphasized the need to reinforce and expand the benefits of public health preparedness and rapid response. On the first page, the GAO report clearly states its purpose: “Because of [the Senate’s] interest in bioterrorism preparedness, you asked GAO to review the public health response to the anthrax incidents” [43].

Finally, President George W. Bush introduced homeland security as the new agenda of the United States government by issuing the Homeland Security Presidential Directive-10 (HSPD-10, or often called to Biodefense for the twenty-first Century) in April 2004. The Homeland Security Act of 2002 was enacted on November 2002, which authorized the establishment of the US Department of Homeland Security (DHS). The homeland security group was deeply involved with the discussion of idea about biodefense as well as the legislation of Project Bioshield Act as seeing the issuance of the Homeland Security Presidential Directive-10 (HSPD-10). The title of the HSPD-10 – *Biodefense for the twenty-first Century* – clearly signs that biodefense was initially subordinate to the homeland security domain. The overall tone of the HSPD-10 is, as the title of the document hints, a security-oriented narrative about defending the US territory and population against biological threats. The main sentence of the HSPD-10 announces that “the United States will continue to use all means necessary to prevent, protect against, and mitigate biological weapons attacks perpetrated against our homeland and our global interests” [44].

##### Biodefense and idea discussed

After the anthrax letter attacks of 2001, common themes of after-action reports and lessons learned analyses emphasized the need for reinforcing and expanding the benefits of “public health preparedness” and the importance of “rapid response” against chemical biological, radiological and nuclear (CBRN) threats [45, 46]. In terms of preparedness and response for national emergencies, particularly bioterrorism events, the mass use of post-exposure prophylaxis (PEP) emerged as key necessity to US biodefense [40]. Vaccines and PEP have quite different medical purposes. A vaccine is an *ex-ante* biological preparation administered before an actual infection in order to provide active acquired immunity to a particular infectious disease, while a PEP is an *ex-post* preventive medical treatment administered after expected exposure to a particular infectious disease in order to prevent becoming infected. During the anthrax letter attacks, an estimated 10,000 individuals, including postal workers, were potentially exposed to *B. anthracis* and advised to take PEPs to prevent inhalational anthrax. However, the US Center for Disease Control and Prevention (CDC) floundered when making a clear decision about the use of prophylaxis. The CDC should mandate specific public health actions, particularly for administration of antibiotic prophylaxis, but there were huge confusions and time-delays surrounding the CDC’s recommendations [47–49]. The United States did not develop emergency response and preparedness measures that strengthen the effectiveness and timeliness of dispensing antimicrobials and vaccines for PEP. Early on, the CDC recommended two antimicrobial prophylaxes – doxycycline and ciprofloxacin – as the post-event countermeasures. However, CDC later selected only doxycycline as a single MCM due to issues regarding efficacy, resistance, side effects, and cost [47].

Moreover, the initial post-exposure prophylaxis (PEP) program recommended 60 days of antimicrobial PEPs (either doxycycline or ciprofloxacin), but later the CDC issued an extended regimen for 40 additional days [47]. The extension was recommended with or without three doses of anthrax vaccine adsorbed (AVA) under an investigational new drug protocol as an extended PEP program [48]. The CDC, as the central federal agency for public health, failed to make timely and appropriate decisions about the use of antibiotic prophylaxis, which caused massive confusion for local-level public health practices during the emergency. Gursky, Inglesby, and O’Toole also point out that it was hard for the CDC as such a research organization to make timely and decisive operational actions at the local level under scientific uncertainties. The key uncertainty in this crisis was the use of post-exposure chemoprophylaxis, for which the CDC struggled to address because it is “a research-based organization, far removed from how public health is delivered” [50].

##### Legislation of the Project Bioshield Act & EUA

The prophylaxis-related issues became the center of lessons learned from the 2001 anthrax letter attack. Most post-event evaluations emphasize that the inefficient coordination between governmental levels resulted in delayed and inappropriate response actions. Particularly, the necessity of a central agency that can perform risk versus benefit-based decision making emerged with the issues relating to the use of prophylaxis. Finally, President George W. Bush signed the Project BioShield Act of 2004 into law, which facilitated the development of MCMs against CBRN agents. The Project BioShield Act was designed to strengthen public health emergency preparedness and response by ensuring the authority of the US government to develop, acquire, stockpile, and make available the medical countermeasures needed to protect the population against WMDs [51]. The implementation of Project Bioshield consists of three major duties: funding needed countermeasures, facilitating research and development, and facilitating the use of MCMs in an emergency; the Emergency Use Authorization (EUA) is one of three main pillars of this Project Bioshield [52]. The US EUA became a legal framework in which the Food and Drug Administration (FDA) is allowed to approve the use of unapproved new MCMs or new off-label indications for previously approved MCMs during a declared emergency.

##### Evolution of the EUA: PAHPA and PAHPRA

The Project Bioshield Act has evolved and revised via the Pandemic and All-Hazards Preparedness Act of 2006 (PAHPA) after the Hurricane Katrina (2005) and the Pandemic and All-Hazards Preparedness Reauthorization Act of 2013 (PAHPRA) after the 2009 H1N1 pandemic. The experience of the Hurricane Katrina (2005) provides the United States the significant lessons to adopt the concept of “all-hazard emergency preparedness” integrating biodefense with public health areas [18]. The 2009 H1N1 global pandemic provided lessons that the US public health preparedness faces a lack of available testing tools as well as countermeasures for emerging infectious diseases [53]. Due to the different public health and security environments, the Bush’s administration’s biodefense strategy has focused on preparing for and responding to public health threats, the Obama’s biosecurity strategy gives the emphasis on prevention efforts [54]. The homeland security domain made by the Amerithrax began to embrace the concept of “emergency preparedness” by the PAHPA of 2006 after Hurricane Katrina and the concept of “disease control and prevention” by the PAHPRA of 2013 after the H1N1 influenza pandemic. Together with these two revisions, the scope of EUA policy broadened from CBRN terrorism threats to other types of threats such as naturally occurring and accidental events.

**Fig. 2:**
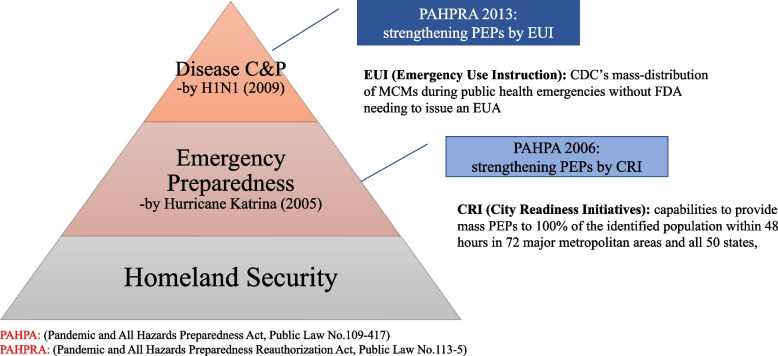
A Structure of the US Biodefense Institutions

Although the scope of the EUA policy expanded in accordance with the PAHPA of 2006 and the PAHPRA of 2013, these two revisions of the EUA policy shared the same path of development with the policy core – the use of unlicensed MCMs as post exposure prophylaxis (PEP) which has kept in the baseline of the newly expanded EUA policies. Under the PAHPA of 2006, two EUA models for doxycycline – the US Postal Service and City Readiness Initiatives (CRI) – were granted. The EUA for doxycycline was combined with mass dispensing models through the US Postal Service and City Readiness Initiatives. These two doxycycline EUA models illustrated that the US biodefense community finally reached an important conclusion from the 2001 anthrax letter attacks: the need to strengthen mass dispensing of PEPs. CRI involves 72 major metropolitan areas and all 50 states, and primarily aims to develop the mass capabilities to provide PEP to 100% of the identified population within 48 h of notification to do so. The United States Postal Service (USPS) is one of the key players in the CRI plan because USPS can deliver antimicrobials (doxycycline hyclate tablets) in the case of an anthrax attack and its medical instructions to residential households within 48 h [55]. Therefore, as seeing Fig. 2, the EUA for doxycycline hyclate tablets, in conjunction with the CRI program, completed the mission of mass and timely distribution of PEPs.

The PAHPA of 2006 was reauthorized by the name of PAHPRA of 2013, and reinforced the mission of mass and timely dispensing of PEPs. The PEP programs for doxycycline such as City Readiness Initiatives are further reinforced by the emergency dispensing order and emergency use instruction (EUI) granted by the PAHPRA of 2013. Both the emergency dispensing order and EUI are advanced forms of the biodefense policy. The FDA explained that the emergency dispensing order authority can “strengthen the nation’s public health protections against CBRN threats by facilitating the availability and use of eligible, approved MCMs needed during public health emergencies without FDA needing to issue an Emergency Use Authorization” [56]. The EUI authority allows the CDC director to facilitate “the availability of streamlined information about the use of eligible, approved MCMs needed during public health emergencies without FDA needing to issue an Emergency Use Authorization” [56].

#### Case study of South Korea

##### Focusing events (2015 MERS) and new agenda (disease containment)

A businessman returning from Bahrain on 4 May 2015 felt sick. Although the businessman visited three different hospitals, no medical professionals suspected that he may have been infected with Middle East Respiratory Syndrome (MERS). The businessman had just returned from a trip to the Middle East; by visiting so many hospitals while the businessman was contagious, he unknowingly infected many healthcare workers and patients with MERS. MERS-CoV, the virus that causes MERS, is a member of the *coronaviridae* family. Same as SARS-CoV-2 causing COVID-19, MERS-CoV features non-specific flu-like symptoms, asymptomatic, and pre-symptomatic transmission, which is hard to identify early. The invisible disease was rapidly spread in Korea by the two amplifiers – nosocomial infection and super-spreader.

First, the MERS outbreak became intensified by nosocomial infection within hospitals [57]. Nosocomial infections, referred to as healthcare-associated infections (HAI), are infections acquired during the process of receiving health care services. In general, hospitals are hubs for sick people who are vulnerable to any kind of infectious diseases. Hospitals unwittingly became the major routes or places of transmission for the 2015 MERS outbreak in South Korea. For example, 85 of the 186 confirmed MERS cases occurred among healthcare workers at Samsung Hospital, the largest general hospital in South Korea. Also, St. Mary’s Hospital in Pyeongtaek, one of the three hospitals visited by patient zero, became the most notorious virus breeding spot infecting 28 people. Second, super-spreaders became another disease amplifier of the MERS outbreak. The businessman (patient zero or index patient) started a chain reaction of disease transmission in multiple hospitals, rendering him a “super-spreader” [58]. This chain reaction of MERS infections further perpetuated transmission as those infected persons sought medical attention at other facilities. The Korea Society of Infectious Disease emphasized the role of five super-spreaders during the MERS outbreak. Case 1 (or patient zero) infected 28 people, case 14 infected 85 people, case 15 infected 6 people, case 16 infected 23 people, and case 76 infected 11 people. These five super-spreaders created 82.3% of the total confirmed cases – 153 cases of 186 total cases [59]. Due to nosocomial infections and super-spreader issues, Korean society descended into chaos; no one knew which hospitals were safe and no one knew who are infected and spread the disease. Containment of the invisible disease spread within society was the first priority for the Korean public health authority.

##### Mobilization of the public health group

The Korea National Assembly established a Special Committee for MERS Prevention in July 2015, which held congressional hearings nine times during the MERS outbreak. The main purpose of the Special Committee was to determine why mass infections were occurring in hospitals and what the ministries responsible for the MERS outbreak did to contain the outbreak. Directors and physicians at the hospitals where the MERS infection had occurred were summoned for hearings where they were asked about the results of epidemiological investigation into mass-infections at their hospitals [60, 61]. Finally, the Special Committee passed a resolution for “reforming national infection prevention and control system” and requested an investigation by the Board of Audit and Inspection (counterpart to the US General Accounting Office) in the Assembly plenary session in August [62]. Based on the Congressional resolution, the Korean government introduced a policy plan, *“Me*asures to Reform National Infection Prevention and Control System for the Purpose of Immediate Response to Emerging Infectious Diseases.” Based on this plan, Korea Center for Disease Control and Prevention (KCDC)’s capabilities and authorities were expanded, and 24-h-a-day Emergency Operation Centers staffed by full-time epidemiologists were created in order to lead the initial response to reports of a new disease outbreak [63].^3^

##### Idea discussed and emergence of disease containment

In 2016, the Ministry of Health and Welfare published the *2015 MERS Outbreak in the Republic of Korea: Learning From MERS*, or simply the “2015 MERS White Paper.” According to this report, the 2015 MERS outbreak was terminated, not by new biomedical technologies, but by traditional disease prevention practices such as epidemiological investigations that identified sick patients who were isolated and exposed individuals who were quarantined [64]. In the absence of medical countermeasures for the treatment or prevention of MERS, non-pharmaceutical interventions (NPIs), such as contact tracing, isolation, and quarantine, became the foundation of South Korea’s public health response. Korea society leaned from the 2015 MERS outbreak that any delay in diagnosing, treating, and isolating an infected patient could unintentionally and unknowingly allow that patient to become a super-spreader. The Korea National Assembly concluded to add Article 34–2 (Disclosure of Information during Infectious Disease Emergency) of the Infectious Disease Control and Prevention Act. This legislation effort implies that accurate and timely diagnostic capabilities are key to identify cases who were infected and who need to be epidemiologically investigated. In other words, diagnostic capabilities are paired with epidemic investigation efforts and epidemic information disclosure policy which becomes the foundation of a new policy – 3 T practice (testing, tracing, and treatment) – later in the COVID-19 pandemic [65]. The Health and Welfare Committee of the National Assembly held a panel discussion on 27 August 2015 on how to reform the public health system to respond more effectively to pandemics. Panelists from government, academia, and private sectors discussed six topics, most of which were related to Korea’s diagnostic capabilities [66]. Also, the Korean Academy of Science and Technology held a round-table discussion with medical professionals about the MERS outbreak and future response plans on 1 July 2015. The participants emphasized the adoption of a US-style EUA policy is essential to identify and trace cases as early as possible [67].

##### EUA legislation in the Medical Device Act

To solve the super-spreader issue, South Korea public health authority adopted EUA policy, officially entitled *The Emergency Use Authorization of In-Vitro Diagnostics for Infectious Disease*. The South Korea government added two clauses regarding the emergency use of diagnostics within “Enforcement Regulations of the Medical Device Act.” Unlike the US EUA legislated in a stand-alone Bill (the Project Bioshield Act), the two clauses (Paragraph 7 of Article 10 and Paragraph 7 of Article 32)^4^ were added in the “Enforcement Regulations of the Medical Device Act” as a legal basis for the emergency use of in-vitro diagnostic kits. According to this law, by commissioner of KCDC, the commissioner of KFDA issues the exemption of testing kit’s examination (authorizing emergency use or called to as EUA) in the case of a public health emergency defined in the Infectious Disease Control and Prevention Act. Because of the legal parameters of the Medical Device Act, the Korean EUA is only applicable to medical devices, such as in-vitro diagnostic (IVD) kits. In contrast to the US approach, which defines MCMs broadly, the Korean EUA cannot issue the use of novel vaccines or therapeutic drugs.^5^

##### Evolution of the EUA: Zika and MERS in 2016, and radioactive contamination

**Fig. 3:**
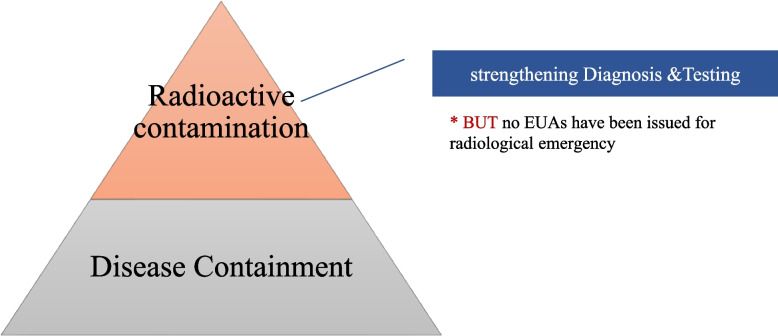
A Structure of the South Korea Biodefense Institutions

Korea’s new EUA policy was first tested in 2016 following the emergence of Zika in South Korea. Among the 14 cases of ZIKV (Zika virus) infection in total from March to October 2016, 9 cases were confirmed by July [68]. On 12 August 2016, the KCDC announced the first issuance of an EUA, which was for MERS diagnostic kits and Zika diagnostic kits. Based on lessons from the 2015 MERS outbreak about the importance of large-scale testing, the Korean public health authority encouraged the private sector to actively participate in testing practice. Same as the purview of the US EUA expanded from bioterrorism to all-hazards, the purview of the Korean EUA also expanded; from infectious diseases to radioactive contamination along with a nuclear crisis in the neighboring country – Japan, as seeing Fig. 3. When a tsunami created a nuclear crisis at Fukushima, Japan in 2011, the world was reminded of the radioactive nightmare of the 1986 Chernobyl disaster. South Korea, as a neighboring country of Japan, paid highest attention to potential radioactivity-related issues and banned the import of Japanese seafood produced by the eight provinces near Fukushima. In May 2015, Japan initiated legal proceedings at the World Trade Organization (WTO), arguing that Korea’s import ban was unreasonable [69].

As the conflict escalated, however, South Korea decided to appeal the ruling and maintain the ban. Also, the Medical Device Act was revised in 2018 to include the threat of a radiological emergency. Instead of legislating a new policy for radiation exposures medications such as iodine or anti-cancer drugs, the purview of the Korean EUA was expanded to include radioactive contamination under the Medical Device Act. The South Korean media raised suspicion that the Korean government aimed to exercise stricter rules for radioactive inspections to all importing products from Japan, as a countermeasure to the Japanese export restrictions [70]. It is worth noting that the Korean EUA was developed along the existing path emphasizing diagnosis (detection), a process of path dependency. Article 46–2 (Special Cases concerning Medical Devices in Cases of Infectious Disease Pandemic) clearly addresses its component of EUA policy that “respond[s] to [an] infectious disease pandemic under the Infectious Disease Control and Prevention Act or radiological emergencies under the Act on Physical Protection and Radiological Emergency” [71].

## Results

In sum, the 2001 Amerithrax and the 2015 MERS outbreak were critical junctures that caused significant changes in biodefense institutions in the United States and South Korea. The US EUA pursued homeland security benefits by focusing on preparedness and response after the 2001 anthrax attacks, while the South Korean EUA pursued public health benefits by focusing on disease containment after the 2015 MERS outbreak. As a result, the US EUA was specialized for mass-treatment practices while the Korean EUA was optimized for mass-diagnosis practices. Through the theoretical lens of historical institutionalism, this study about EUA policy proposes that the two critical junctures (2001 Amerithrax and 2015 MERS outbreak) were decisive moments resulting in institutional innovation in the two countries. The US EUA is the one of the representative post-Amerithrax phenomena leading to the emergence of a new institution—homeland security-oriented biodefense—in the US. The Korean EUA was also created as part of a broader biodefense strategy to contain infectious diseases based on the country’s experience with the 2015 MERS outbreak.

In addition to the role of exogenous shocks as critical junctures, these case studies demonstrate the actual role of endogenous factors when it comes to institutional changes. The path-dependency narrative has explained the evolutionary path of institutional changes in a broad sense. This article contributes to determining what really happens at the domestic level when institutions are changing by walking on the same pathway of development as their previous innovations. The emergence of the new policy domains in these two countries indicates a key linkage between exogenous and endogenous factors in the establishment of biodefense systems. The homeland security group in the US and the epidemiology group in South Korea were mobilized in the aftermath of the focusing events. As a result, the US biodefense policies (e.g., EUA) that would once have been considered public health priorities were developed and implemented primarily in the context of homeland security; this perception underscores the issuance of EUA for post-prophylaxis (PEP) use of unapproved medical countermeasures with broader efforts to “prepare for and respond” to the threat of CBRN terrorism. On the other hand, Korean policymakers and public health authorities perceive the EUA as a tool for disease containment against emerging infectious diseases; this perception underscores Korea’s issuance of EUA for non-pharmaceutical intervention (NPI) use of unapproved medical countermeasures for “detection and diagnosis,” of radiological contamination. In other words, PEP represents the core of the US biodefense that has been strengthened through “self-reinforcement” under the homeland security domain despite subsequent policy revisions made in response to focusing events such as Hurricane Katrina or H1N1 influenza. In the same vein, the Korean biodefense NPI core has been fortified under the disease containment domain and later subsumed radiological contamination in the list of targeted threats.

## Discussion and conclusion

There are two main implications of this study for the global health community. First, this article determines how institutions, especially in the context of public health and biodefense institutions, are created and revised. Basically, the meso-level lens from historical institutionalism could provide an analytical framework in the context of critical juncture and path dependency on the abstract level. Moreover, Birkland’s Event-related Policy Change Model provides only a descriptive narrative about what drives policy changes. Building upon the two theories with mutual complementarity, this article contributes to determining the dynamic between exogenous and endogenous factors and their effect on institutional changes. Specifically, it examines how exogenous shocks result in the emergence of domestic coalitions that become endogenous forces for policy revision in a self-reinforcing manner.

In addition to the above-mentioned theoretical contribution, this article illustrates the difference in the features of biodefense institutions in the US and South Korea. It discusses how EUA policies became crucial after the COVID-19 outbreak for the facilitation of counter-pandemic measures, and even though the US already had an EUA policy, it suffered from highly inadequate COVID-19 testing in the early phase of the pandemic. As US House Oversight Committee Chairwoman Carolyn Maloney pointed out, the US had tested only 425 cases by 25 February, while South Korea had tested over 35,000 cases. She raised a question during a congressional hearing: Why are we [the US] so far behind South Korea in testing and reporting this crisis? [72]. This article can provide an answer: The Korean EUA was oriented toward mass-testing in response to infectious disease outbreak, but the US EUA was designed with the intention to provide mass-treatment in response to CBRN threats.

This article introduced the origination, purpose, and evolutionary paths of institutional changes and demonstrated its applicability to the study of how EUA policies, as a representative of the biodefense institutions, undergo changes in the US and South Korea. Further research is needed on how the EUAs really worked during the COVID-19 pandemic;

What are the features of EUAs in other countries that newly legislated EUA during the COVID-19 pandemic?

Understanding the correlation between exogenous and endogenous factors in terms of institutional changes would contribute significantly to enhancing global public health and improving health security against future public health threats.

## Data Availability

No applicable.

## Abbreviations

CBRN: Chemical, Biological, Radiological, and Nuclear
CDC: US Center for Disease Control and Prevention
COVID-19: pandemic caused by the new corona virus (SARS-CoV-2)
CRI: City Readiness Initiatives
DHS: US Department of Homeland Security
EUA: Emergency Use Authorization
FDA: US Food and Drug Administration
GAO: US General Accounting Office
HI: Historical Institutionalism
IVD: In-Vitro Diagnostic kit
KCDC: Korea Center for Disease Control and Prevention
KFDA: Korea Food and Drug Administration
MCMs: Medical Countermeasures
MERS: Middle East Respiratory Syndrome
NPI: Non-Pharmaceutical Intervention
PAHPA: Pandemic and All-Hazards Preparedness Act of 2006
PAHPRA: Pandemic and All-Hazards Preparedness Reauthorization Act of 2013
PEP: Post-Exposure Prophylaxis
USPS: The United States Postal Service

## Footnotes

1 MCMs (Medical Countermeasures): consists of biologics (vaccines), therapeutic drugs and other medical devices that may be used in public health emergencies.

2 Japan also has Emergency Approval (EA) policy similar to EUA, but operational mechanisms between EA and EUA are quite different such as eligible products and authority of emergency declaration.

3 KCDC was promoted to Agency-level organization during the COVID-19 pandemic (September 2020). Now the official name of this organization is Korea Disease Control and Prevention Agency (KDCA).

4 Both clauses were deleted on 31 December 2018. Instead, Article 46–2, newly inserted a “Medical Device Act” by Act No. 15486, 13 March 2018, which becomes a new legal basis of the Korean EUA.

5 The Korean EUA policy was revised in March 2021 to open the list of eligible products from diagnostic kits only to all MCMs including all biologics such as vaccines and therapeutic drugs.
